# Decontamination of organic pollutants from aqueous media using cotton fiber–graphene oxide composite, utilizing batch and filter adsorption techniques: a comparative study

**DOI:** 10.1039/c8ra10449b

**Published:** 2019-02-18

**Authors:** A. I. Abd-Elhamid, A. A. Nayl, Ahmed A. El. Shanshory, Hesham M. A. Soliman, H. F. Aly

**Affiliations:** Advanced Technology and New Materials Research Institute, City for Scientific Research and Technology Applications, SRTA P. O. Box 21934 Egypt; Chemistry Department, College of Science, Jouf University Sakakah Saudi Arabia aanayl@yahoo.com; Hot Laboratories Center, Atomic Energy Authority Nasr 13759 Egypt

## Abstract

Cotton fiber–graphene oxide (C–GO) composite with high adsorptive properties towards the cationic dye, crystal violet (CV), was successfully fabricated by simple mixing of cotton fiber and GO in aqueous solution using a homogenizer. The as-prepared composite was characterized using TEM, SEM, LOM, XRD, FTIR, Raman and TGA. The characterization indicated that the formation of a homogeneous composite occurred *via* adequate mixing of the cotton fiber and GO. The fine structure of the obtained composite was successfully used in two adsorption techniques, namely batch adsorption and filter adsorption. Various parameters affecting batch adsorption, such as contact time, dye concentration, composite dose, NaCl dose, temperature and pH were investigated. In the filter adsorption mode, dye concentration, composite dose, NaCl dose, temperature, flow rate and pH were studied. A comparison study between the two techniques, *i.e.*, batch adsorption and filter adsorption, are reported. The filter adsorption technique shows higher adsorption efficiency than the batch one, which was evident from the maximum adsorption capacity (*Q*°) values, obtained from the Langmuir isotherm. Further, the filter technique was developed and evaluated. This was achieved by regeneration, scaling-up and, finally, using another model of cationic dye (methylene blue).

## Abstract 2

Cotton fiber–graphene oxide (C–GO) composite with high adsorptive properties towards the cationic dye, crystal violet (CV), was successfully fabricated by simple mixing of cotton fiber and GO in aqueous solution using a homogenizer.

## Graphical abstract

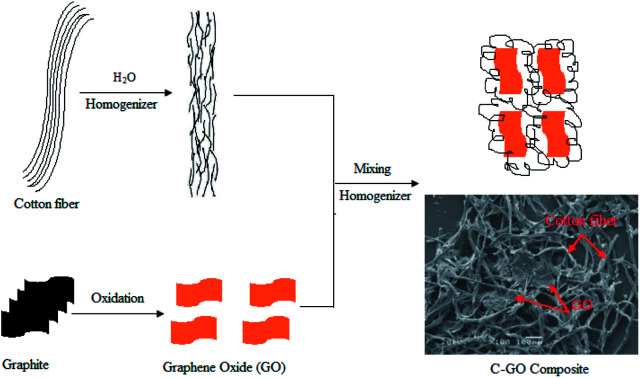

## 1. Introduction

Discharge of dye containing effluents to the environment is harmful to various kinds of living organisms. Cationic dyes (methylene blue and crystal violet), anionic dyes (methyl orange and eriochrome black T) and/or azo dyes (acid red 1 and acid red 40) may be present in the dye effluents. Annually, several hundred thousand tons of dye stuff are produced.^1–4^ Many essential industries, such as textile, paper, and plastic, use dyes to color their products. Moreover, these industries require a huge amount of water. Hence, a significant quantity of colored wastewater effluent should be treated before it is released into the environment for both toxicological and esthetical reasons.^5^

Several approaches, including physical, chemical and biological, have been studied by researchers to remove dyes from waste. These methods suffer from some drawbacks, such as the formation of by-products, release of aromatic amines, methane and hydrogen sulfide production as a result of anaerobic breakdown, high cost, and the requirement for a lot of dissolved O_2_. Adsorption is one of the methods, applied to treat wastewater, which is considered to be an efficient and superior tool in many cases for wastewater treatment technology.^6^ This method may be desirable owing to its low initial cost, simplicity of design, ease of operation and lack of harmful exhaust.

Because of its unique structure, GO is estimated to be one of the more powerful adsorbents. GO monolayer is one C-atom thick, and it is decorated with a large number of oxygen-rich active groups, such as OH, on the basal plane and COOH edges.^7^

Cotton is a suitable substrate material due to its high surface area, mechanical support and low weight. Numerous techniques have been investigated for creating hybridization between GO and the cotton fiber, such as dipping and drying,^8,9^ layer by layer coating,^10^ dipping,^11^ and brush-coating and drying.^12^ However, these methods are time consuming, and they require a multi-step preparation. Also, they produce GO-layers which coat the fiber surface, and GO-monolayers cannot penetrate the fiber.

The present study deals with preparation of a low cost adsorbent with a high tendency to adsorb a cationic dye form an aqueous solution. The composite was prepared *via* a one-pot reaction using a homogenizer by simply mixing the cotton fiber and GO in an aqueous solution.

In detail, the homogenizer allows the cotton fiber, pumped at high pressure, to pass through a small gap inside it. These gaps are created by a valve and an impact ring. When working, the valve is rapidly opened and closed. This subjects the cotton fibers to high shear and impact forces, which provoke cellulose fiber nanofibrillation. By introducing GO into the homogenization media, monolayers get in between the cotton nanofibers. Hence, the GO monolayers will be distributed overall the cotton fiber used. Moreover, this method provides a link between the cotton fiber and GO, preventing disintegration of the composite during the treatment process.

Additionally, the main problem with using GO is the placement of its monolayers, which are compacted upon drying. In this state, most of the active groups on the both sides of the layer will be hindered from the adsorbed species, and it will take a time for them to be available for the adsorption process. In this study we assume that the GO-monolayers will be separated by the cotton nanofiber. As a result, GO will keep its efficiency of adsorbing the pollutant species. On the other hand, the removal of GO from the treated media appears to be a difficult process due to its high dispensability, but strong interaction between GO and the cotton fiber makes the isolation process easier. The soft structure of the obtained composite was found suitable for use in various adsorption techniques. Therefore, two techniques for removal of the cationic dye from water were studied: batch adsorption and filter system. A comparison between the two techniques was performed and discussed.

## 2. Experimental

### 2.1. Materials and laboratory equipment

All chemicals were of analytical grade and used as received. These were Crystal Violet (CV) and Methylene Blue (MB) (99%, Sigma-Aldrich), sulfuric acid (95–97%, Riedel de Haen), potassium permanganate (99%, Long live), hydrogen peroxide (36%, Pharaohs Trading and Import), graphite (200 mesh, 99.99%, Alpha Aesar), potassium persulfate (≥99.0%, Sigma-Aldrich), hydrochloric acid (30%, El Salam for Chemical Industries), and cotton (from the local market).

The following equipment was used: homogenizer (T18 Basic, Ika, Germany), water distillatory (2108, GLF, Germany) for double distilled water, pH meter (3510, Genway), hot plate stirrer (SB 162, Stuart, UK), centrifuge, (Mikro 220R, Hettich, UK), UV/Vis Spectrophotometer-Double beam (T80+, PG instruments Ltd., UK), and analytical balance (CP 2245, Sartorius, USA).

### 2.2. Preparation of GO and cotton–GO composite

GO was prepared as described by Ji Chen *et al.*^13^ with some modifications. Briefly, to 70.0 mL concentrated sulfuric acid solution in 150 mL glass beaker 3.0 g graphite powder was added under stirring for 10 minutes. This suspension was kept at 20 °C in a water bath and 9.0 g KMnO_4_ was gradually added, followed by gradual addition of 3.0 g potassium persulfate. The temperature of this mixture was raised to 40 °C under vigorous stirring for about 30 min. The slurry was added to 150 mL double distilled water and the solution was stirred for 15 min at 95 °C. The volume of the final slurry was increased to 500 mL using double distilled water, followed by a slow addition of 15 mL H_2_O_2_ (30%). The color of the suspension turned from dark brown to yellow. The mixture was filtered and the filtrate of the prepared GO was washed with 250 mL of 10% HCl aqueous solution. Finally, the GO product was washed with 250 mL double distilled water and dried at 35 °C for 99 h. The resultant powder was used for further experiments.

**Fig. 1:**
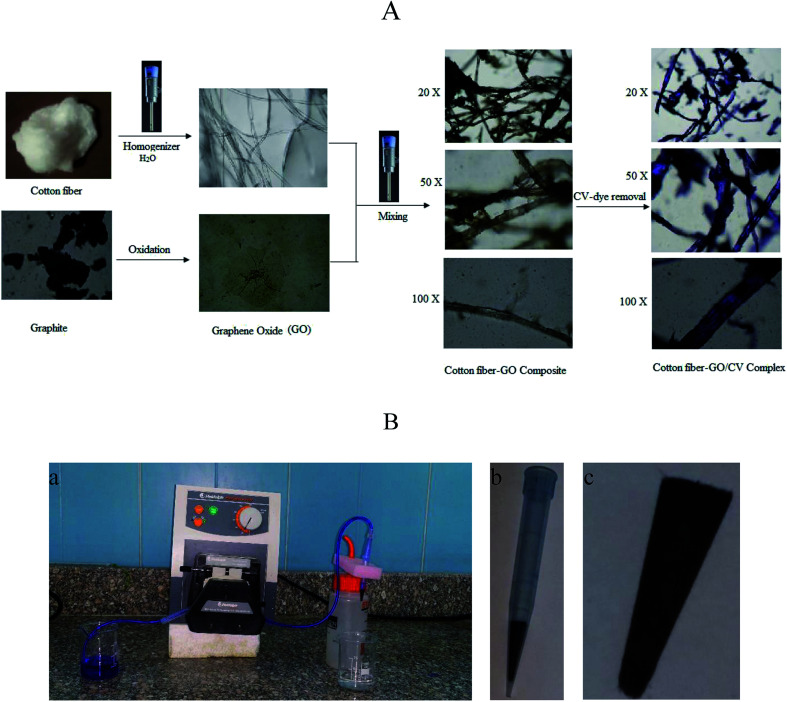
(A) Schematic depicting images (at different magnifications) of cotton fiber, GO, brown cotton–GO composite and violet composite after the treatment; images were obtained using an optical microscope at various experimental stages. (B) Photographic images of (a) the filter adsorption equipment, (b) 1 mL plastic tip loaded with C–GO composite and (c) the composite shape after packing inside the tip.

The cotton–GO composite was prepared by dispersing 10.0 g of cotton fiber in 500 mL double distilled water using homogenizer. To this suspension 0.5 g of the prepared GO powder was added under homogenization until complete mixing between the cotton fiber and GO was achieved. Finally, the solution of cotton–GO composite was removed by filtration and dried at 70 °C for 24 h. Schematic diagram of optical images explaining the different stages of preparation of C–GO composite and the composite after the adsorption process are presented in Fig. 1A.

### 2.3. Characterization

#### 2.3.1. Transition electron microscopy (TEM)

Transmission Electron Microscopy (TEM) images were obtained using TEM (JEOL GSM-6610LV, Japan) instrument at the accelerating voltage of 200 kV. The materials were suspended in an aqueous solution using ultrasonication. A micropipette was used to transfer a drop of the suspended material to a carbon grid. Finally, the grid was air dried and used for characterization.

#### 2.3.2. Scanning electron microscopy (SEM)

The cotton and cotton–GO composite surface morphologies were determined using a scanning electron microscope (SEM, JEOL GSM-6610LV, Japan), operating at acceleration voltage of 20 kV. Specimen surfaces were coated with a thin layer of gold before observation. SEM images were obtained at different magnifications. Elemental analysis was carried out using the EDS unit.

#### 2.3.3. Light optical microscopy (LOM)

The optical images (at different magnifications) of GO, cotton fiber, cotton–GO composite and the composite after the treatment process were obtained using Light Optical Microscopy (LOM) (BX61, Olympus, Japan).

#### 2.3.4. X-ray diffraction (XRD)

The crystal structures of GO, cotton fiber and cotton–GO composite were determined using X-ray diffraction (XRD, Shimadzu, Japan XRD-7000) with a scanning speed of 12° min^−1^ from 5° to 100°.

#### 2.3.5. Fourier transform infrared spectroscopy (FT-IR)

Infrared spectra of GO, cotton fiber, cotton–GO composite, CV-dye and cotton–GO composite after the adsorption of the CV-dye were obtained using a Fourier transform infrared spectrometer (FTIR, Shimadzu FTIR-8400 S, Japan). Experiments were carried out in the range of 4000 to 400 cm^−1^.

#### 2.3.6. Raman spectroscopy

Raman spectra were obtained using a Raman Microscope (Bruker, Senterra II, Germany) with an excitation wavelength of 514 nm and power of 5 mW.

#### 2.3.7. Thermo-gravimetric analysis (TGA)

Thermal stability of GO, cotton fiber, and cotton–GO composite was characterized using Thermo-Gravimetric Analyzer (Shimadzu Thermal Gravimetric Analysis (TGA)—50, Japan). All measurements were performed under a nitrogen atmosphere with a flow rate of 10 mL min^−1^ by heating the material from 25 °C to 600 °C at a heating rate of 10 °C min^−1^.

### 2.4. Batch adsorption of CV

One g L^−1^ of CV-dye solution was prepared as a stock solution and the desired concentrations, needed for the subsequent studies, were prepared by further dilution using double distilled water. The effect of adsorption time on CV-dye removal was studied by adding 0.03 g of the prepared composite to a 50.0 mL solution containing 10 mg L^−1^ of the dye at pH 7.0 and 25 °C at known time intervals until equilibrium was reached. Known concentrations of the dye solution in the range (10–50 mg L^−1^) were used to study the effect of the initial dye concentration. Certain known weights of the composite (0.01–0.05 g) were added to 50.0 mL of CV-dye solutions with concentration of 30 mg L^−1^. The initial solution pH range was adjusted to 2.5–9.5 using 0.5 mol L^−1^ HCl or NaOH. Salinity test was performed by adding different amounts (0.0–0.5 g) of NaCl to 30 mg L^−1^ of the dye solution. The effect of the temperature on the adsorption process was investigated in the range of 20–80 °C. The obtained liquid samples were isolated by centrifugation and CV concentration was determined using UV-spectrophotometer at the wavelength of 590 nm and in case of MB at 662 nm. The dye removal efficiency (% *R*) is defined as1
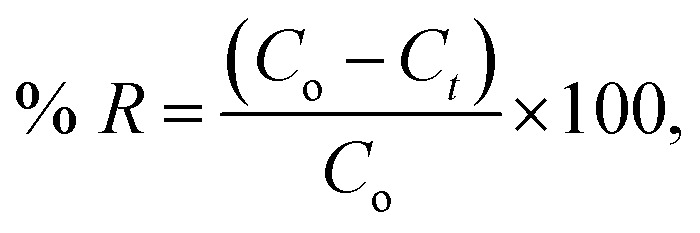
where *C*_o_ and *C*_*t*_ are the initial concentration of the dye and the concentration of the dye at time *t*, respectively.

### 2.5. Continuous removal of CV

The equipment used for the continuous removal process contained a plastic pipe. One end of this pipe was used for withdrawing the dye solution (input). The other end was connected to a plastic tip (1.0 mL) of a micropipette. This plastic tip was loaded with different known amounts of the prepared composite (0.01–0.05 g) and served as the outlet for the treated water, as illustrated in Fig. 1B.

The removal of CV-dye from the aqueous solution using the filter system was carried out by passing the solution of the dye through the packed composite. The treatment experiments were performed by immersing of 50 mL of the CV solution of various concentrations (10–50 mg L^−1^) in a 100 mL glass beaker. Composite dose in the range of 0.01–0.05 g was used and the concentration of the dye supplied was 30 mg L^−1^. The effect of salinity was tested by adding NaCl in the range of 0.0–0.50 g to the 30 mg L^−1^ dye solution at different temperatures and variant flow rate in the pH range of 2.5–9.50. Finally, 0.5 mL of the output solution was diluted to 5 mL by double distilled water, and CV concentration was determined as previously described. The dye removal efficiency (% *R*) was determined using eqn (1).

### 2.6. Regeneration experiments

To regenerate the composite for further use, a 10 mL of 4% HCl solution was passed through the packed filter, followed by 5 mL of distilled water for washing, 10 mL of 20% EDTA-aqueous solution and, finally, 5 mL of distilled water for washing with a flow rate of 0.625 mL min^−1^ by the peristaltic pump. The removal percentage of the dye in each cycle was calculated using eqn (1).

## 3. Results and discussion

### 3.1. Composite characterization

#### 3.1.1. TEM, SEM and LOM

**Fig. 2:**
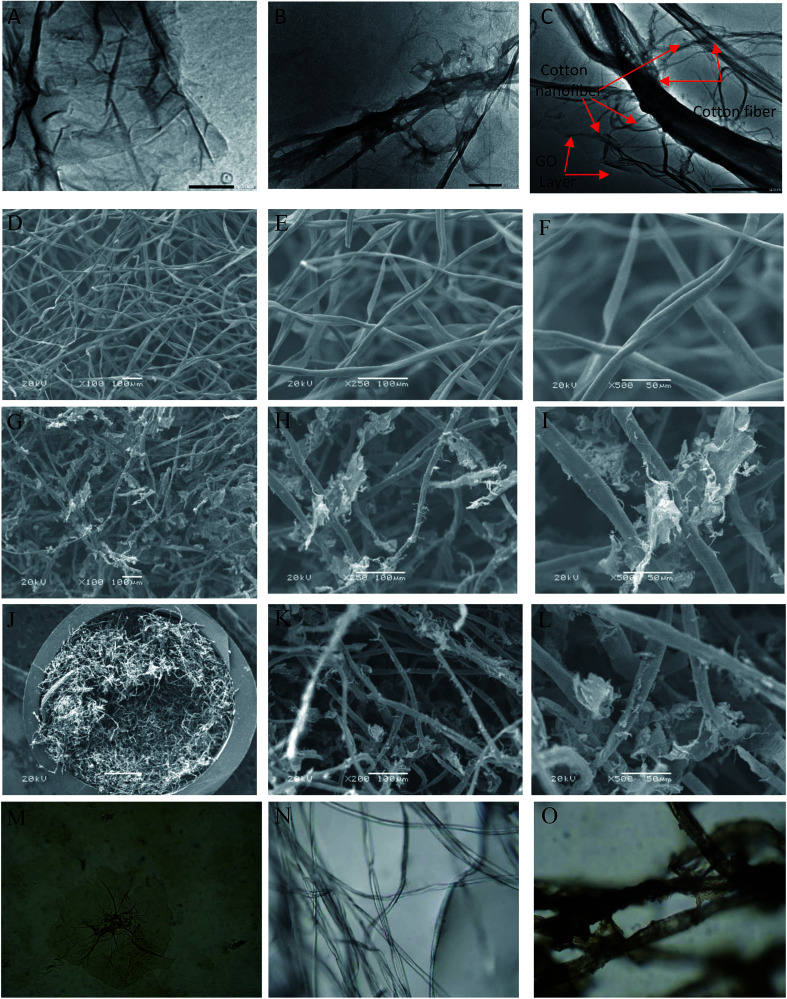
(A) TEM image of GO, (B and C) TEM image of C–GO composite, (D–F) SEM image of the cotton fiber, (G–I) SEM images of the C–GO composite, (J–L) SEM images of C–GO packed in the filter, (M) LOM images of GO sheets, (N) LOM images of the cotton fiber, and (O) LOM images of C–GO composite.

The surface morphology of GO, cotton and cotton–GO (C–GO) composite was investigated using TEM, SEM and LOM. As shown in Fig. 2A, the sheets of GO appear to contain two-dimensional flakes. For cotton–GO composite, Fig. 2B and C, it is clear that the cotton fibers were covered with the GO sheets which keep on the exfoliation of GO-sheets and increase the reactivity of the composite.

The SEM images, presented in Fig. 2D–F, show the cotton fiber photographs at different magnification. They indicates that the cotton fiber has a uniform size and a smooth surface. On the other hand, by mixing GO and the cotton fiber, we can see that the GO-sheets are well mixed with the cotton fiber (Fig. 2G–I). This observation is also supported by Fig. 2J–L, which contain the SEM images of C–GO composite packed in the filter system at different magnifications. In addition, in the LOM image, Fig. 2M, GO-nanosheets appear as a light brown layer.^7,14^ The cotton fiber preformed as a white-colored fiber, Fig. 2N. Finally, the optical image of the C–GO composite shows a brown fiber of the cotton–GO complex, Fig. 2O.

#### 3.1.2. X-ray diffraction

**Fig. 3:**
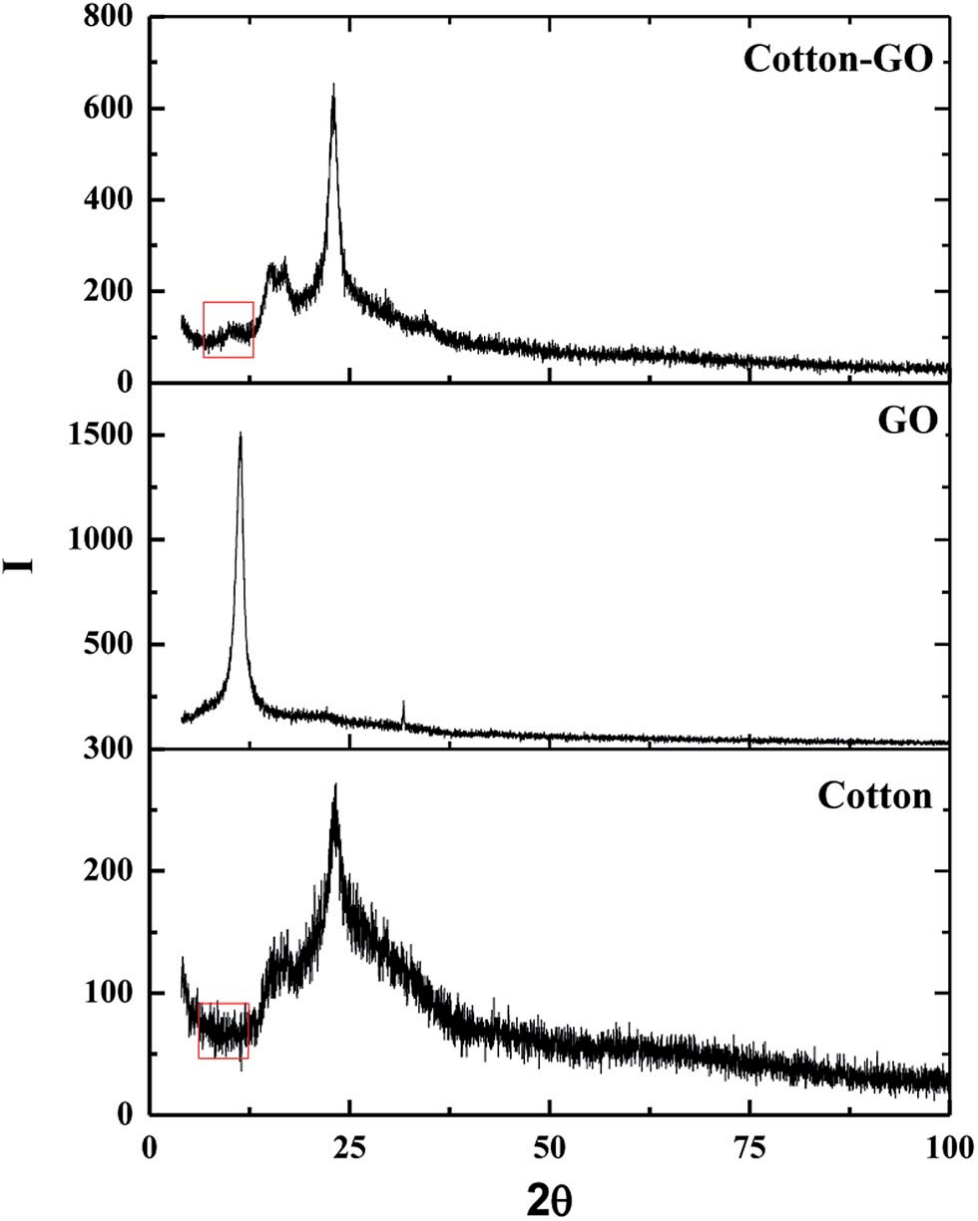
X-ray diffraction pattern of cotton, GO and cotton–GO composite.

The crystallinity of cotton, GO and C–GO composite was analyzed using X-ray diffraction, as presented in Fig. 3. Cotton had a sharp diffraction peak at 2*Θ* = 23° which was consistent with the crystal structure of cotton.^15,16^ Since the concentration of GO in the composite was low, the characteristic GO peak at 2*Θ* = 11.2° (see Fig. 3) was very weak in the prepared C–GO composite,^11^ as seen in Fig. 3. Moreover, the diffraction peaks of C–GO composite pattern at 2*Θ* = 15°, 16° become more visible. The peak at 2*Θ* = 23° appears to be sharper and more intense compared with the corresponding peak of the native cotton fiber. This is because the fluffy staple cotton fiber converted to a fine structure upon formation of composite.

#### 3.1.3. FTIR analysis

**Fig. 4:**
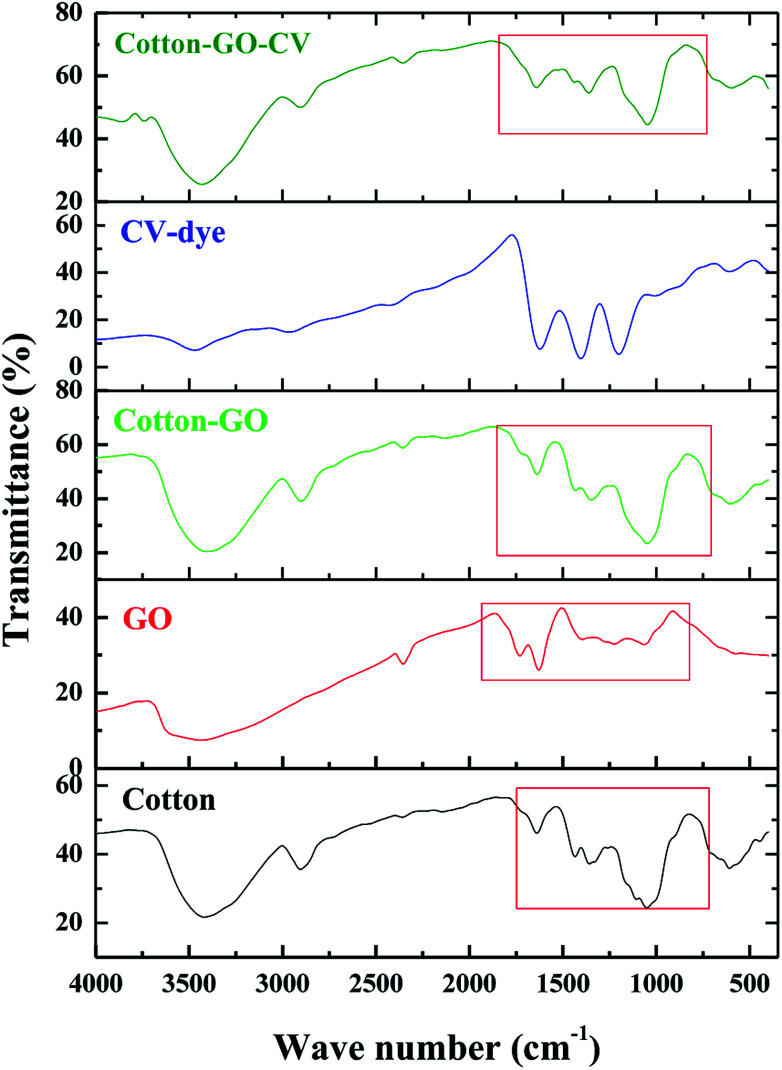
FTIR spectra of cotton, GO, cotton–GO composite, CV-dye and composite–CV complex.

FTIR spectra in the range 400–4000 cm^−1^ of cotton, GO, cotton–GO, CV-dye and cotton–GO-CV are found in Fig. 4. The spectrum of cotton contains bands at 3419 cm^−1^, 2904 cm^−1^, 1639 cm^−1^, 1435 cm^−1^, 1359 cm^−1^, and 1051 cm^−1^, which correspond to O–H stretching vibration, C–H stretching vibration, O–H bending vibration, C=O stretching vibration, O–H deformation^11^ and alkoxy C–O stretching vibration, respectively. The characteristic GO bands represent O–H stretching vibration at 3433 cm^−1^, COOH group at 1730 cm^−1^,^17^ O–H bending vibration at 1629 cm^−1^, O–H deformation vibration at 1394 cm^−1^ and alkoxy C–O stretching vibration at 1064 cm^−1^. Cotton–GO composite, Fig. 4, exhibits absorption bands at 3406 cm^−1^ corresponding to O–H stretching of the adsorbed water, C–H stretching vibration at 2901 cm^−1^, COOH group shoulder at 1730 cm^−1^, O–H bending vibration at 1637 cm^−1^, and C–O stretching vibration at 1049 cm^−1^. Additionally, the bands characteristic of the CV-dye are seen in Fig. 4. When the CV-dye was adsorbed onto the C–GO composite, the bands at 3406 cm^−1^, 2901 cm^−1^, 1637 cm^−1^, 1348 cm^−1^, and 1049 cm^−1^ shifted to 3431 cm^−1^, 2904 cm^−1^, 1641 cm^−1^, 1435 cm^−1^, 1359 cm^−1^, and 1047 cm^−1^, respectively. These results confirmed the adsorption of the dye onto the composite.

#### 3.1.4. Raman spectra

**Fig. 5:**
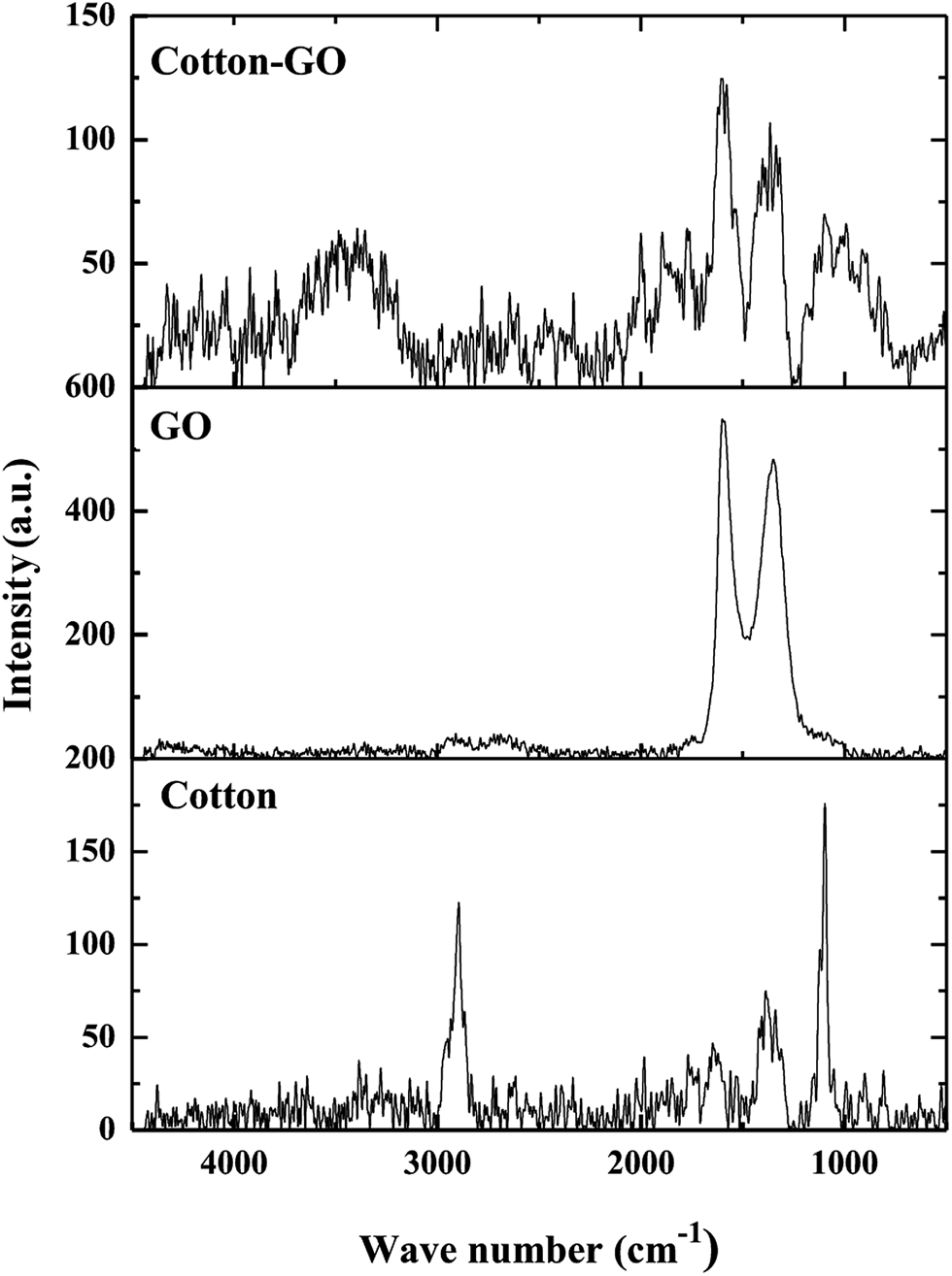
Raman spectra of cotton, GO and cotton–GO composite.

Raman spectroscopy is widely used to analyze structures of carbon materials. Raman spectra of cotton, GO and C–GO composite are displayed in Fig. 5. The cotton fiber spectrum contains bands at 2897 cm^−1^, 1366 cm^−1^ and 1099 cm^−1^, which correspond to stretching vibration of C–H and –CH_2_,^18^ a symmetric stretching vibration of –CH_2_ (ref. 19) and stretching vibration of C–O–C glycosidic band,^20^ respectively. Furthermore, D-band at 1340 cm^−1^ and G-band at 1614 cm^−1^, characteristic of GO,^21^ are shown in Fig. 5. The broadening of these bands, G-bands and D-bands, shows the degree of disorder in graphite. On the other hand, the ratio of the of D-band intensity, *I*_D_, to the G-band intensity, *I*_G_, represents the sp^2^/sp^3^ carbon ratio. Moreover, the Raman spectrum of the C–GO composite exhibits a shift in the position of D and G-bands to 1363 cm^−1^ and 1604 cm^−1^, respectively, comparing with GO spectrum (Fig. 5). The positions of the D and G bands and *I*_D_/*I*_G_ ratio for GO and C–GO composite were calculated and listed in Table 1. Variation in the *I*_D_/*I*_G_ value of 1.71 for GO to 1.28 for C–GO may refer to the interaction that takes place between GO and cotton.

**Table 1:** The D and G-band positions, *I*_D_/*I*_G_ ratios and FWHMs

Sample	D-band peak		G-band peak		*I*_D_/*I*_G_
	Raman shift (cm^−1^)	FWHM (cm^−1^)	Raman shift (cm^−1^)	FWHM (cm^−1^)	
GO	1340	137	1614′	80	1.71
C–GO	1363	148	1604	116	1.28

#### 3.1.5. TGA analysis

**Fig. 6:**
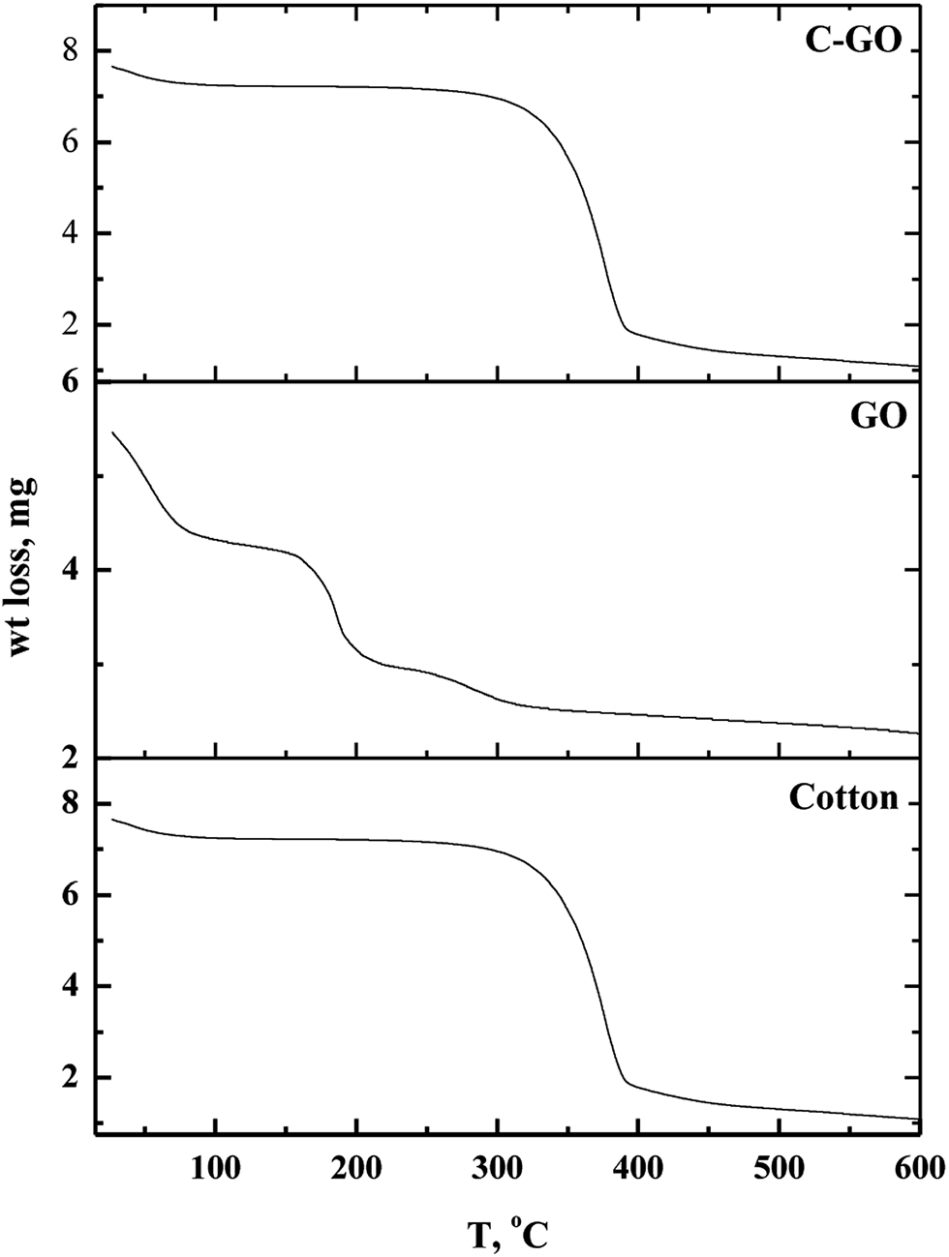
Thermo-gravimetric diagrams of cotton, GO and cotton–GO composite.

Thermo-gravimetric analysis is an effective way to determine the weight loss temperature, weight loss rate and the residual weight of the analyzed sample. Thermal stability of cotton, GO and cotton–GO composite was investigated, and the data are shown in Fig. 6. At temperatures below 286 °C, cotton fiber shows a gradual weight loss, which corresponds to dehydration of cotton fiber.^22^ The maximum pyrolysis of the cotton fiber initiated at 286 °C and terminated at 400 °C. This stage involves degradation of saccharide rings and molecular chain fractionation.^23^ Above 400 °C the degradation rate was slow until the temperature reached 600 °C; the residual weight of the cotton was 2.31%. On the other hand, GO decomposed into four stages, shown in Fig. 6: evaporation of moisture at 28–88 °C, dehydration of adsorbed water at 88–158 °C, pyrolysis of O-rich functional groups –OH and C–O–C at 158–215 °C, and, finally, decomposition of –COOH at 215–320 °C. The addition of GO to the cotton fiber produced a small variation in the thermogram data of the obtained composite compared with that of the cotton fiber, as shown in Fig. 6. For example, weight loss below 286 °C was 7.75% for cotton and 11.325% for C–GO. Extensive degradation began at 286 °C for cotton and 293 °C for C–GO. The residual weight was 2.31% for cotton and 2.1% for C–GO.

### 3.2. Adsorption process

#### 3.2.1. Effect of contact time

**Fig. 7:**
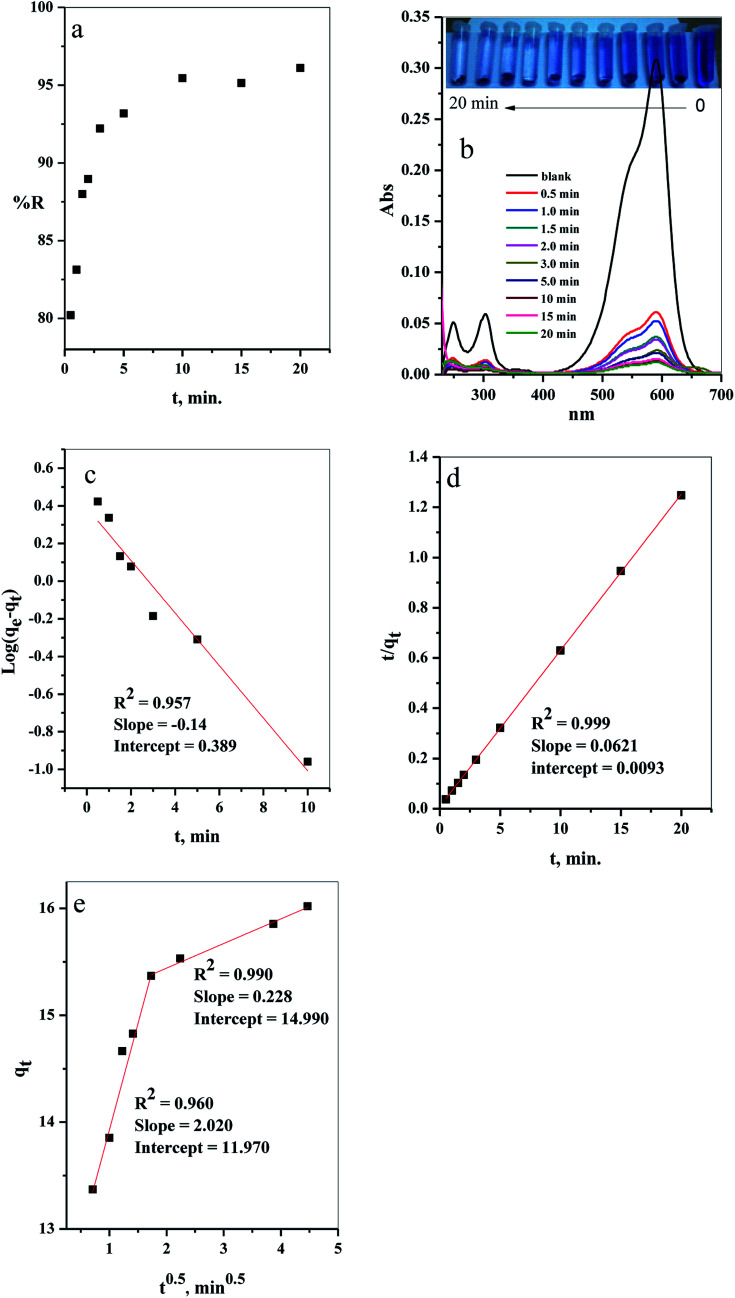
The effect of contact time on the (a) removal percent (% *R*) from aqueous solution, (b) UV-Vis measurements of the maximum absorption peak of CV at several time intervals (inset depicts the optical image of CV-dye solution at different contact times), (c) pseudo first-order model, (d) pseudo second-order kinetic model and (e) intraparticle diffusion model. Batch → C–GO dose = 0.03 g, CV = 10 mg L^−1^, solution volume = 50 mL and *T* = 25 °C.

The results of evaluation of the contact time, required for removal of CV-dye from aqueous solution using a batch adsorption experiment, are presented in Fig. 7a. It is evident that CV-dye quickly adsorbed onto the composite; the equilibrium was reached within 10 minutes and % *R* = 95.45. The UV-Vis spectrum of CV-dye removal at different stirring times is presented in Fig. 7b. The maximum absorption peak at 590 nm gradually decreased as the contact time increased, as shown in Fig. 7b. A photographic image of the samples isolated with the time variation from 0.0–20.0 min is shown in the inset the Fig. 7.

##### 3.2.1.1. Adsorption kinetics

The adsorption kinetics of the CV-dye adsorption process can be explained using pseudo first order (eqn (2)),^24^ pseudo second order (eqn (5))^25^ and intraparticle diffusion models (eqn (6))^26^ and illustrated in Fig. 7c–e, respectively.2
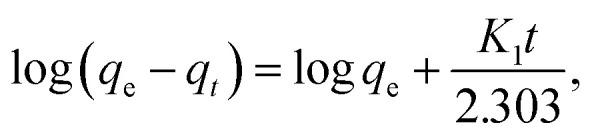
where *q*_e_ (mg g^−1^) is the amount of sorption at equilibrium time, *q*_*t*_ (mg g^−1^) is the amount of sorption at time *t* and *K*_1_ (min^−1^) is the rate constant of pseudo first order sorption.3
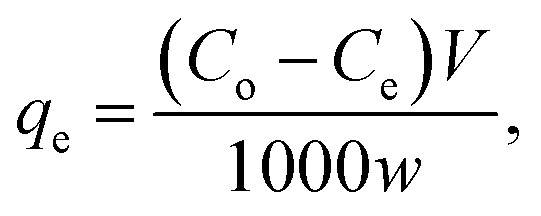
where *C*_o_ is the initial concentration (mg L^−1^), *C*_e_ is the dye concentration at equilibrium time intervals (mg L^−1^), *V* is the volume of dye solution (mL) and *w* is the mass of adsorbent (g).4
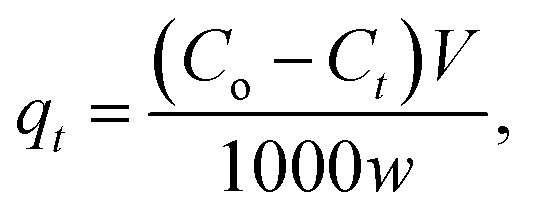
where *C*_*t*_ is the dye concentration at different time intervals (mg L^−1^).5
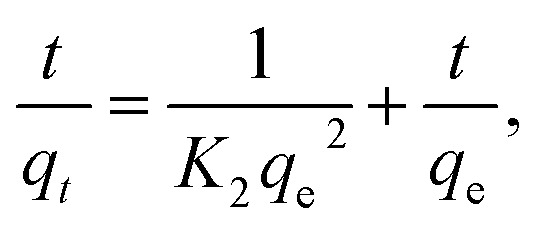
where, *K*_2_ (g mg^−1^ min^−1^) is the rate constant of pseudo second order reaction

6 *q*_*t*_ = *k*_i_*t*^0.5^ + *C*,

where *k*_i_ is the intra-particle diffusion rate constant (mg g^−1^ min^−0.5^), *q*_*t*_ is the amount of dye adsorbed (mg g^−1^), and *C* is the intercept.

The kinetic parameters of the experiments, such as *q*_e exp_, *K*_1_, *q*_e cal_, *R*^2^ and *K*_2_, were calculated using Fig. 7c and d and listed in Table 2. The results demonstrate that the adsorption of CV-dye onto the composite follows the pseudo second order. This phenomenon is attributed to the fact that the amount of crystal violet, adsorbed at equilibrium and calculated using the pseudo second order (*q*_e cal_ = 16.10 mg g^−1^), is closer to the adsorbed amount, calculated using experiment data (*q*_e exp_ = 16.0 mg g^−1^). Moreover, the correlation coefficient (*R*^2^ = 0.999), which is related to pseudo second order model, is larger than the one related to pseudo first order model (*R*^2^ = 0.957).

**Table 2:** Calculated parameters of the pseudo first-order and pseudo second-order kinetic models of batch

Dye	*q*_e exp_ (mg g^−1^)	First-order kinetic parameter			Second-order kinetic parameter		
		*K*_1_ (min^−1^)	*q*_e cal_ (mg g^−1^)	*R* ^2^	*K*_2_ (g mg^−1^ min^−1^)	*q*_e cal_ (mg g^−1^)	*R* ^2^
CV	16.02	−0.322	2.450	0.957	0.415	16.103	0.999

Furthermore, various stages of adsorption process were analyzed, applying the intraparticle diffusion model (Fig. 7e). The adsorption process contained two adsorption stages. The first one involved the diffusion of the dye-molecule on the external surface of the composite. The second stage was the gradual adsorption stage.

#### 3.2.2. Effect of initial dye concentration

**Fig. 8:**
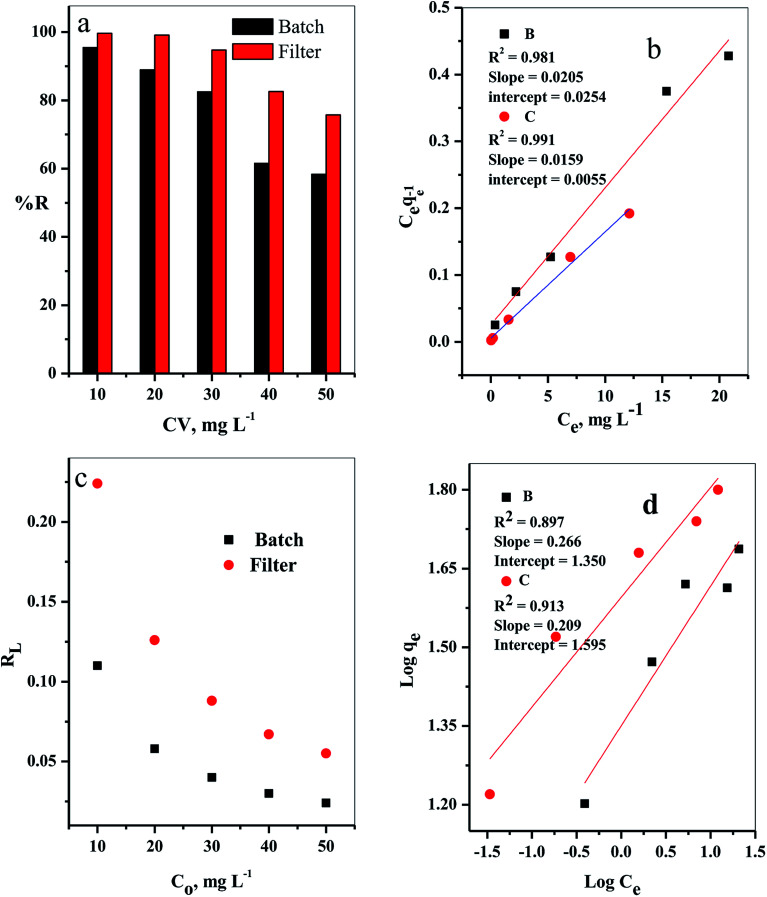
The effect of initial CV-dye concentration for both batch (B) and filter (C) techniques on (a) % *R*, (b) Langmuir isotherm model, (c) separation factor (*R*_L_) and (d) Freundlich isotherm model. Batch → contact time = 10 min, C–GO dose = 0.03 g, solution volume = 50 mL and *T* = 25 °C. Filter → C–GO dose = 0.03 g, solution volume = 50 mL, *T* = 25 °C and flow rate = 2.78 mL min^−1^.

The influence of the initial dye concentration on the removal percentage (% *R*) of the CV-dye from aqueous solution was investigated for both batch and filter techniques, as presented in Fig. 8a. At a low initial dye concentration (10 mg L^−1^), the removal percentage values were % *R* = 95.45 for batch and 99.66 for filter techniques. Further increase of the initial dye concentration to *C*_o_ = 50 mg L^−1^ led to a gradual decrease of the removal percentage values to % *R* = 58.37 for batch and 75.75 for filter methods. This phenomenon could occur because at low concentration of the dye species, the number of available active sites was sufficient for adsorption of all the dye species, whereas at a high dye concentration, the number of active sites was insufficient for of adsorption all the dye species.^7^

It is important to indicate that the filter shows higher ability to remove the CV-dye from the aqueous solution than batch adsorption, as clarified in Fig. 8a.

##### 3.2.2.1. Adsorption isotherm

The adsorption isotherm models were studied by Langmuir^27^ and Freundlich^28^ isotherm, eqn (7) and (8), respectively.7
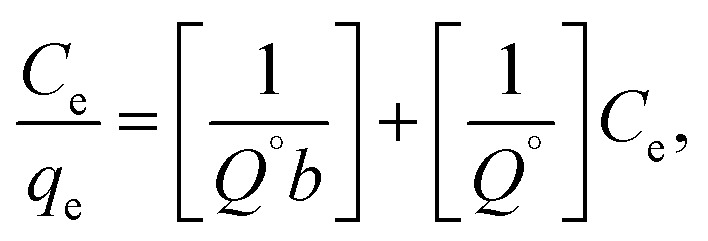
where *q*_e_ is the amount of solute adsorbed per unit weight of adsorbent (mg g^−1^), *C*_e_ is the equilibrium concentration of the solute in the bulk of solution (mg L^−1^), *Q*° is the monolayer adsorption capacity in mg g^−1^ and *b* is the constant related to the free energy of adsorption. A plot of *C*_e_/*q*_e_*versus C*_e_ gives a straight line with *Q*° and *b* determined from the slope and the intercept, respectively.8
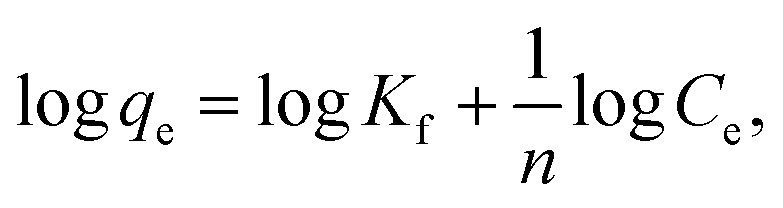
where *K*_f_ in mg g^−1^ and *n* are Freundlich constants, related to adsorption capacity and adsorption intensity, respectively.

The results, obtained from the experimental data, were plotted in Fig. 8b–d, and the isotherm parameters were calculated and recorded in Table 3. When using Langmuir isotherm, it is assumed that the dye molecules adsorb homogeneously onto the active sites, and no further adsorption takes place at that site. The Langmuir model, related to this study (Table 3), has high correlation coefficients, *R*^2^ = 0.981 and 0.991 for batch and filter, respectively, which suggest homogenous adsorption of CV-dye onto the C–GO composite surface. The monolayer maximum adsorption capacity, *Q*°, and *b* for batch are 48.780 mg g^−1^ and 0.810 L mg^−1^, whereby for filter, they are 62.890 mg g^−1^ and 0.346 L mg^−1^. On the other hand, Freundlich model assumes that the active sites located on the adsorbent surface sites have inequivalent binding energies, and the powerful binding sites are occupied first. Furthermore, the Freundlich constant, 1/*n*, values for batch (0.266) and filter (0.210) imply favorable adsorption of CV-dye on the C–GO composite (Table 3). The high values of *K*_f_, Table 3, indicate that the C–GO composite shows high adsorption capacity for CV-dye in aqueous solution.

**Table 3:** Calculated equilibrium constants for adsorption of CV on composite

Technique	Langmuir isotherm model			Freundlich isotherm model		
	*Q*° (mg g^−1^)	*b* (L mg^−1^)	*R* ^2^	1/*n*	*K*_f_ (mg g^−1^)	*R* ^2^
Batch	48.780	0.810	0.981	0.266	22.420	0.900
Filter	62.890	0.346	0.991	0.210	39.252	0.916

In addition, the dimensionless constant *R*_L_,^29^ separation factor, values corresponding Langmuir isotherm is less than the unit, *R*_L_ < 1, as explained in Fig. 8c, and show favorable adsorption.^30^ Finally, the correlation coefficients, corresponding to the Langmuir model are higher than those corresponding to the Freundlich model (Table 3). This observation indicated that the adsorption of CV-dye onto the C–GO composites as a monolayer using batch adsorption or filter adsorption is favorable.

Moreover, the maximum adsorption capacity of various adsorbents, utilized for the removal of CV-dye,^31–37^ and the results of the current study are summarized in Table 4. The present comparison shows that *Q*° values obtained in this study are higher than those obtained for the reported adsorbents.

**Table 4:** Adsorption capacities of different adsorbents for CV-dye

Adsorbent	*Q*° (mg g^−1^)	Ref.
Jute fiber carbon	27.99	32
Coniferous pinus bark powder	32.78	33
TiO_2_-based nanosheet	58.30	34
Moroccan pyrophyllite	9.58	35
NaOH-modified rice husk	44.876	36
*Punica granatum* shell	50.21	37
*Artocarpus heterophyllus* (jackfruit) leaf powder (JLP)	43.39	38
PAN/β-CD/GO	16.47	45
CNF-based PVDF membrane	2.948	46
Meldrum's acid modified CNFbased PVDF membrane	3.984	46
WCNF	48	47
Batch adsorption	48.780	This study
Filter adsorption	62.890	This study

#### 3.2.3. Effect of cotton–GO composite dose

**Fig. 9:**
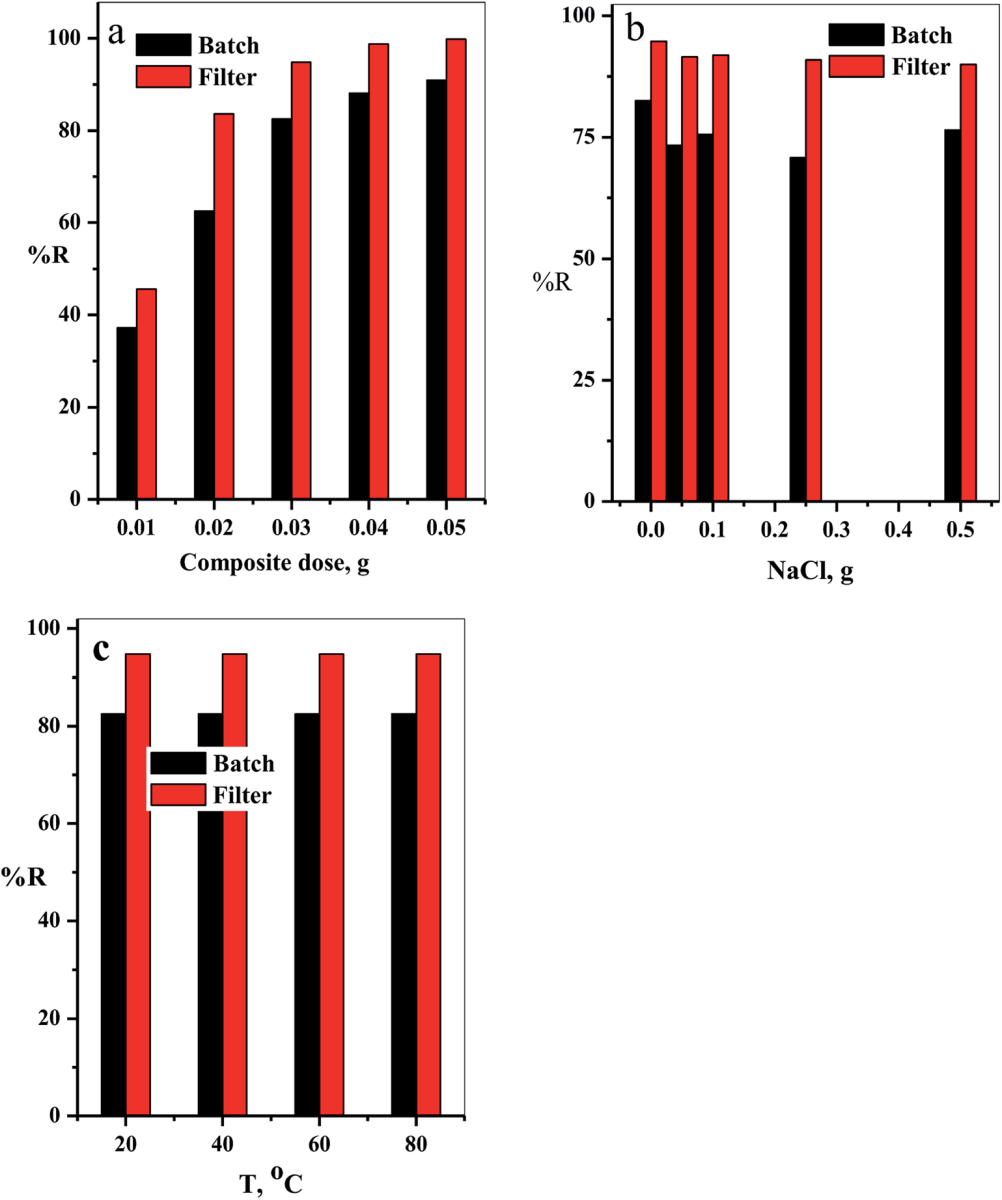
(a) The effect of the composite dose, (b) NaCl dose and (c) temperature on % *R* of CV-dye in aqueous solution for both batch and filter techniques. Batch → contact time = 10 min, CV = 30 mg L^−1^ and solution volume = 50 mL. Filter → CV = 30 mg L^−1^, solution volume = 50 mL and flow rate = 2.78 mL min^−1^.

The relation between the initial composite dose, g, and the removal percentage, % *R*, was studied in the range 0.01 to 0.05 g for both batch and filter techniques and explained in Fig. 9a. The related results demonstrate that the % *R* increases with the increasing adsorbent dose up to 0.05 g. The main reason for this result is the increase in number of the available active sites for adsorption of the dye species as the adsorbent dose increases. This leads to an increased number of the attached dye species and, consequently, increased % *R* value.^38^

#### 3.2.4. Effect of NaCl dose

Variation of removal percentage of the CV-dye upon addition of different doses of NaCl in the range of 0.0–0.5 g for both batch and filter techniques is demonstrated in Fig. 9b. The experimental data show that as the weight of NaCl increases, the removal process exhibits a slight variation in % *R*. This phenomenon may explain as follows. Under experimental conditions, the electrostatic attraction between the negatively charged composite and the positively charged CV-dye species is not significantly affected by the addition of NaCl.

#### 3.2.5. Effect of solution temperature

The effect of temperature on the % *R* of CV-dye in aqueous solution for both batch and filter techniques was investigated in the temperature range of 20–80 °C, as illustrated in Fig. 9c. The data show that there is no variation in the % *R* with a further increase in temperature within the range studied. This result is in a good agreement with our previous study.^7^

#### 3.2.6. Effect of solution flow rate

**Fig. 10:**
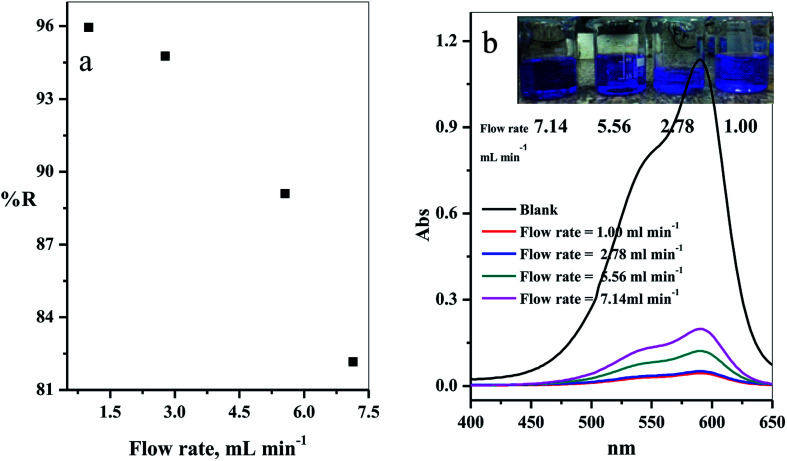
(a) Effect of the flow rate of CV-dye solution on the removal percentage (using a filter) and (b) UV-Vis spectra of the maximum absorption peaks of CV at different flow rates; inset contains an optical image of CV-dye solution at different flow rates. Filter → C–GO dose = 0.03 g, CV = 30 mg L^−1^, solution volume = 50 mL and pH = 7, *T* = 25 °C.

The effect of the flow rate in mL min^−1^ on adsorption of CV-species in aqueous medium was investigated. The experimental results obtained are displayed in Fig. 10a. Four different flow rates 1.00, 2.78, 5.56 and 7.14 mL min^−1^ were examined. As expected, the removal percentage decreased with increased flow rate (Fig. 10a). This happened because the high flow rate resulted in insufficient contact time to reach the equilibrium state between the dye species and active sites in the filter.^39–41^

The intensity of the maximum absorption peak increased as the flow rate increased, shown in Fig. 10b, and the intensity of violet color increased as seen in the photo-image inset of Fig. 10b.

#### 3.2.7. Effect of initial solution pH

**Fig. 11:**
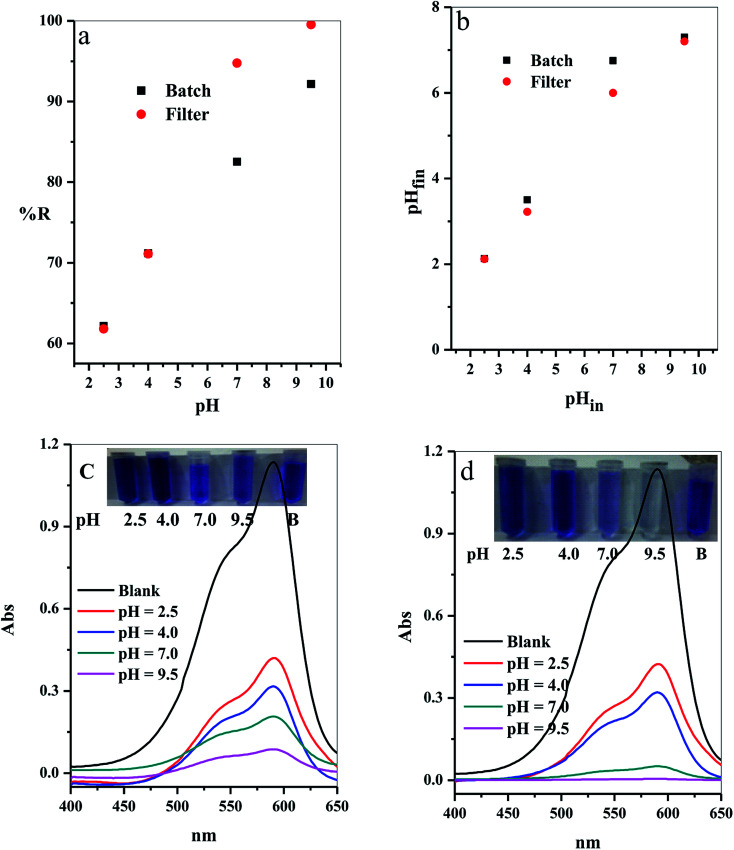
Effect of the initial solution pH on (a) The removal percentage (% *R*) from aqueous solution, (b) Final pH of the treated solution for batch (B) and filter (C) techniques and (c and d) UV-Vis spectra of the maximum absorption peaks of CV at several time intervals; inset shows optical image of CV-dye solution at different initial pH values for batch and filter techniques, respectively. Batch → contact time = 10 min, C–GO dose = 0.03 g, CV = 30 mg L^−1^, solution volume = 50 mL and *T* = 25 °C. Filter → C–GO dose = 0.03 g, CV = 30 mg L^−1^, solution volume = 50 mL, *T* = 25 °C and flow rate = 2.78 mL min^−1^.

The adsorption process in aqueous medium was highly influenced by the initial hydrogen ion concentration, pH.^42^ This commonly agree with the pH at which values do not affect both the adsorbate surface active sites and also the adsorbent dye species. Variation of the % *R* with the different initial pH values (2.5–9.5) was examined as shown in Fig. 11a. The results indicated that the adsorption of CV-dye for both batch and filter was highly depended on the initial pH of aqueous solution,^43^ where at pH = 2.50, the % *R* = 62.16 (for batch) and 61.80 (for filter), and at pH = 9.50, % *R* = 92.16 (for batch) and 99.55 (for filter).

#### 3.2.8. Mechanism of the adsorption

The prepared composite carries OH and COOH groups that act as active sites for adsorption of the dye molecules. Hence, at low pH values, the adsorbent possesses low % *R* towards the CV-dye, caused by protonation of the active site by the high concentration of protons, [H^+^]. These protons compete with the cationic CV-dye for the available active sites.^44^ On the contrary, at high pH values, the active site is completely ionized, increasing the number if negative charges on the adsorbent. Thus, the electrostatic attraction between the active sites and the dye increases, consequently, increasing % *R*.

The relation between the initial pH_in_ and the final pH_fin_ of the dye solution is shown in Fig. 11b and the calculated values are listed in Table 5. The analysis of the data, obtained from Fig. 11b and Table 5, indicated that the initial pH decreased over the entire pH range. Moreover, we observed that the highest decrease in the pH_in_ occured in the alkaline medium, where % *R* exhibits the highest value (Table 5). These results support the above suggested mechanism, which indicates that adsorption of the dye molecules causes liberation of H^+^ ions in the solution. This leads to a decrease in the pH_in_ value and the ΔpH value increases as a result of the increasing % *R*.

**Table 5:** Values of pH_in_, pH_fin_, ΔpH and related % *R* for adsorption of CV onto composite

Technique	pH_in_	pH_fin_	ΔpH = pH_in_ − pH_fin_	% *R*
Batch	2.5	2.13	0.37	62.16
	4	3.50	0.50	71.17
	7	6.75	0.25	82.523
	9.5	7.30	2.20	92.16
Filter	2.5	2.13	0.37	61.8
	4	3.22	0.80	71.1
	7	6.00	1.00	94.77
	9.5	7.20	2.30	99.55

The optical images and UV-Vis spectra of the CV-dye samples, separated at different pH values after the treatment process using batch technique and filter technique, are presented in Fig. 11c and d. They support the experimental results in terms of the variation in the color of the treated solutions.

#### 3.2.9. Regeneration and reusability

**Fig. 12:**
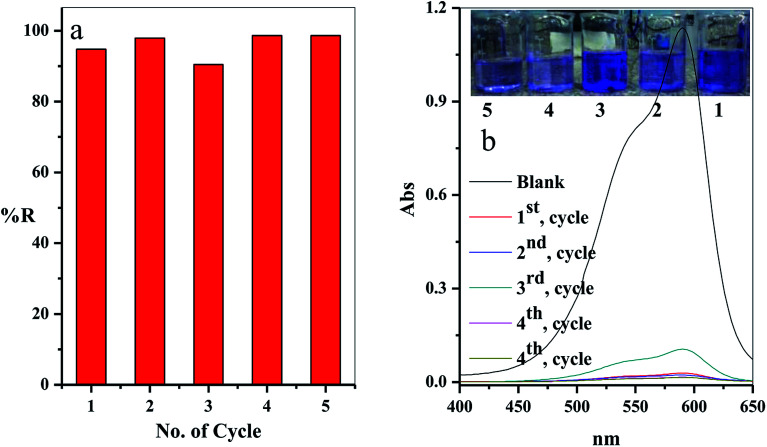
(a) The effect of the number of the re-use cycles of the filter on the removal percentage of CV-dye solution and (b) UV-Vis spectra of the maximum absorption peaks of CV at different reuse cycles; inset shows optical image of CV-dye solution at different cycles. Filter → C–GO dose = 0.03 g, CV = 30 mg L^−1^, solution volume = 50 mL, pH = 7, *T* = 25 °C and flow rate = 2.78 mL min^−1^.

From the economic point of view, it is essential for desorption and regeneration step of the used adsorbent. In this study, the adsorbent filter was regenerated by passing the regenerated solution through the filter using the peristatic pump. The adsorption-regeneration step was repeated five times with approximately the same removal percentage of the CV-dye, as presented in Fig. 12a. The concentration of the remaining CV-dye in the treated sample was determined using a UV-Vis spectrophotometer, as shown in Fig. 12b. Inset of Fig. 12b shows a photographic image of the treated dye solution.

#### 3.2.10. Scaling up

The scale-up of the filter adsorption technique was carried out to make our experiment more applicable and efficient. For this purpose, 1.5 L of the contaminated water solution containing 10 mg L^−1^ CV-dye at pH = 7 and temperature 25 °C was prepared. This dye-contaminated water was divided into six equal portions of 250 mL. Each portion was passed through the same packed cotton–GO filter containing 0.20 g at the flow rate of 2.78 mL min^−1^. The concentration of the CV-dye in each treated portion of water was determined using UV-Vis spectrophotometry at 590 nm. Furthermore, a second model of cationic dye, methylene blue (MB), was treated under similar conditions, used for CV-dye. In this case, the UV-Vis spectrophotometry was used to determine the residual concentration of the MB-dye solution at 662 nm.

**Fig. 13:**
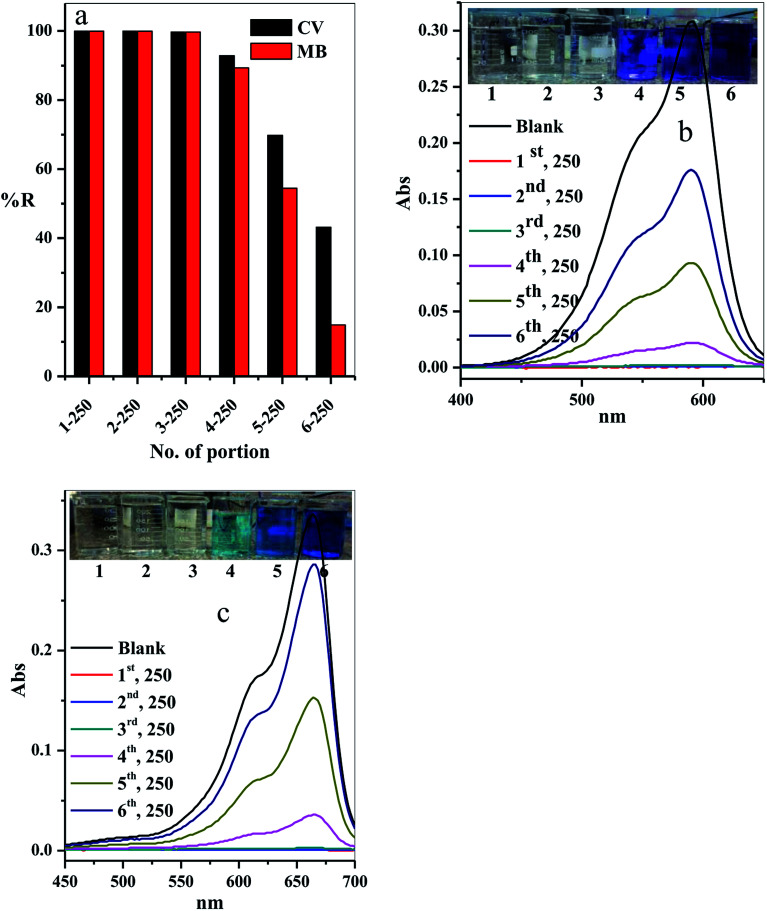
Effect of number of portions on the (a) removal percentage of the CV and MB dyes, (b and c) acquired for the supernatant liquids collected and the absorption spectra for CV and MB at different portion number during dye adsorption experiments. Filter → C–GO dose = 0.20 g, CV = MB = 10 mg L^−1^, solution volume = 1.50 L, pH = 7, *T* = 25 °C and flow rate = 2.78 mL min^−1^.

The residual concentration in both treated dye solutions (crystal violet and methylene blue) was plotted as a function of the number of the treated portions, as shown in Fig. 13a. It is clear that the dyes (crystal violet and methylene blue) were completely removed from the first three portions (% *R* = 99.90%). The % *R* gradually decreased in the last three portions to 92.86, 69.81 and 43.18, respectively, for crystal violet dye and to 89.29, 54.46 and 14.88, respectively, for methylene blue. These results can be observed by the liquids collected and the absorption spectra for crystal violet (Fig. 13b) and methylene blue (Fig. 13c) for various portion number during dye adsorption experiments.

#### 3.2.11. Characterization of the composite after the adsorption

**Fig. 14:**
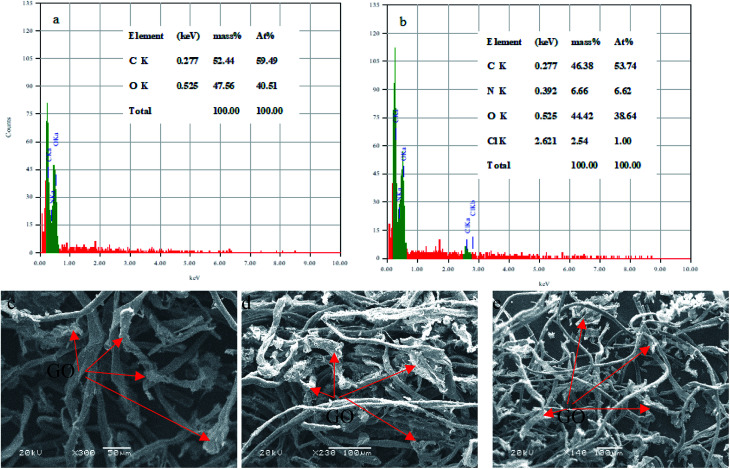
EDS analysis of (a) C–GO composite and (b) C–GO-CV dye. SEM images of the C–GO composite after adsorption (c–e).

The EDS analysis was used to determine the elemental C–GO composition of the composite before and after the adsorption process, as demonstrated in Fig. 14a and b, respectively. Here, we use the EDS analysis to confirm the adsorption of the dye onto the composite. The analysis demonstrated that the pure composite consisted of carbon (59.49%) and oxygen (40.51%). On the other hand, the composite after the treatment process contained nitrogen (6.62%) and chloride (1%), presence of which confirm the adsorption of the dye. Moreover, SEM was used to estimate the stability of the composite after the adsorption process, as seen in Fig. 14c–e. The obtained SEM images from different three areas shows clearly that the GO is very well tightly with the cotton fiber, as shown in Fig. 14c–e.

## 4. Conclusion

C–GO composite was successfully prepared and used for the adsorption of cationic dye from the aqueous solution. Batch adsorption and filter adsorption were performed in this study. The filter adsorption process achieved better removal of CV-dye with *Q*° = 62.89 mg g^−1^, which is higher than *Q*° = 48.78 mg g^−1^ in batch adsorption. This phenomenon may be attributed to the fact that in the filter, all the dye species in the solution are forced to contact active sites, increasing the removal efficiency. The filter in our system is easy to regenerate and its production can be scaled up, enhancing its application on industrial scale.

## Conflicts of interest

There are no conflicts to declare.

## Supplementary Material
